# *Piriformospora indica* culture filtrate and cell extract induce chicoric acid production in *Echinacea purpurea* hairy roots

**DOI:** 10.1371/journal.pone.0323961

**Published:** 2025-06-17

**Authors:** Samane Khalili, Ahmad Moieni, Naser Safaie, Mohammad Sadegh Sabet

**Affiliations:** 1 Department of Plant Genetics and Breeding, Faculty of Agriculture, Tarbiat Modares University, Tehran, P.O. Box: 14115-336, Iran; 2 Department of Plant Pathology, Faculty of Agriculture, Tarbiat Modares University, Tehran, P.O. Box: 14115-336, Iran.; Shiraz University, IRAN, ISLAMIC REPUBLIC OF

## Abstract

*Echinacea purpurea* (L.) Moench, commonly known as purple coneflower, is a significant medicinal plant renowned for its therapeutic properties, which are attributed to various phytochemical compounds, including caffeic acid derivatives (CADs). Chicoric acid is one of the CADs that has important immunostimulatory properties. This study employed a hairy roots (HRs) culture and an elicitation system to enhance the production of chicoric acid in *E. purpurea*. HRs cultures were established, and different concentrations (0, 1.25, 2.5, 5, and 10% *v/v*) of elicitors derived from *Piriformospora indica* culture filtrate (CF) and cell extract (CE) were added at two time points during the HRs growth period (on days 24 and 26). The effects of these treatments on the growth of HRs, chicoric acid production, and the expression of genes involved in the chicoric acid biosynthesis pathway were investigated. The highest dry weight of HRs (2.19 g/L, 1.36% higher than that in the control) was achieved in the HRs culture treated with 5% CF on the 24th day. In contrast, 5 and 10% (*v/v*) of *P. indica* CE, regardless of addition time, significantly decreased HRs growth compared to the control. The maximum production of chicoric acid (15.52 mg/g DW) was recorded after 48 h in the HRs culture treated with 5% CE on day 24, representing a 2.6-fold increase compared to the control (5.95 mg/g DW). Additionally, adding 2.5% CF to the HRs culture on day 26 resulted in a 2.3-fold increase compared to the control (13.5 mg/g DW) in chicoric acid biosynthesis. Real-time PCR assays revealed that the expression levels of the genes *PAL*, *C4H*, *4CL*, *C3H*, and *HCT* were significantly upregulated after 3 and 12 h of elicitation with CE and CF. The highest gene expression was recorded for the *C4H* and *PAL* genes, 3 h after elicitation by CE (29.64 and 26.2-fold increases compared to the control culture). In contrast, the expressions of the *4CL* and *C3H* genes peaked 12 h after elicitation with CF. The expression of the *HCT* gene also reached its highest level after 12 h of CE elicitation. Consistent with the chicoric acid production results, CE was found to be a more effective elicitor for inducing gene expression in the chicoric acid biosynthesis pathway. Overall, these findings indicate that HRs cultures and elicitors derived from *P. indica* are promising strategies to enhance chicoric acid production in *E. purpurea* (L.).

## Introduction

Secondary metabolites are a group of organic compounds produced as part of a plant’s defense mechanism in response to environmental stresses. These compounds help protect plants from insect attacks, pathogens, and other stresses. They also exhibit various biological activities and are used in many industries, including dyes, food processing, cosmetics, and pharmaceuticals [1,2]. *Echinacea purpurea*, commonly known as purple coneflower, is one of the most important medicinal plants of the Asteraceae family and is native to the Midwestern United States [3]. The global demand for this plant has increased significantly due to its secondary metabolites and phytochemical compounds, prompting its cultivation worldwide [4]. The primary secondary metabolites found in *E. purpurea* include flavonoids (such as luteolin and quercetin), phenylpropanoids (like caffeic acid and its derivatives), terpenoids (like borneol and germacrene D), glycosides (such as echinacoside and echinacin), and nitrogen-containing compounds, including alkamides and alkaloids [5]. These compounds are used to treat colds, respiratory issues, and urinary diseases due to their immunological, antiviral, antifungal, and antibacterial properties [6–10]. One of the important derivatives of caffeic acid is chicoric acid, which has demonstrated immunostimulatory, antitumor, and anti-inflammatory properties. It also promotes phagocytic activity under both *in vitro* and *in vivo* conditions [11]. Furthermore, chicoric acid exhibits anti-hyaluronidase activity and offers protection against collagen degradation caused by free radicals [12]. Five critical enzymes in the phenylpropanoid pathway are essential for the synthesis of chicoric acid: phenylalanine ammonia-lyase (PAL), cinnamate 4-hydroxylase (C4H), 4-coumarate coenzyme A (CoA) ligase (4CL), coumarate 3′-hydroxylase (C3H), and shikimate O-hydroxycinnamoyl transferase (HCT) [13]. The content of active compounds can vary significantly from plant to plant and year to year due to contamination of plant materials with insects, fungi, bacteria, and soil, as well as a lack of pure and standardized plant materials. Researchers are exploring various methods to enhance the production of secondary metabolites. Plant cell and hairy root (HRs) cultures have emerged as promising alternatives for producing metabolites that are challenging to obtain through chemical synthesis or traditional plant extraction methods [14,15]. Using various strategies, including the selection of HRs culture, the use of stimulants for cultivation, and optimizing culture medium conditions, can help overcome some limitations in economically producing secondary metabolites from this valuable medicinal plant [16]. The HRs culture is a potential system for commercial purposes due to its ability to synthesize secondary metabolites even more effectively than intact plants, cell suspensions, and callus cultures [17]. The rapid growth in hormone-free culture medium, genetic and biochemical stability, and the ability to produce various secondary metabolites offer several advantages for HRs culture [18]. Using elicitor, both biotic and abiotic, is a common method for increasing secondary metabolite production in cell culture systems [19]. Elicitors stimulate the production of secondary metabolites by activating transcription factor synthesis and regulating gene expression of biosynthetic pathways. [20,21]. In addition, increased antimicrobial activity has been reported in plant extracts treated with elicitors [22]. Microorganisms, especially fungi and their compounds, have been employed to boost secondary metabolite production in agricultural ecosystems due to their low cost, high efficiency, and minimal toxicity to plant cell cultures [23,24]. The active compounds in fungal extracts can induce plant defense responses. Enzymes that degrade cell walls, such as cellulose and xylanase, along with compounds like flavonoids, oligosaccharides, hormones, and peptides found in fungal extracts, can act as elicitors [25]. *Piriformospora indica,* discovered in India in 1997, is a symbiotic mycorrhizal-like fungus that possesses a broader host range among mono- and dicotyledonous plants. It has been used as a model to study the mechanisms and evolution of mutualistic symbiosis [26,27]. Researchers have reported the importance of this symbiotic relationship in enhancing plant growth and yield, as well as improving plant tolerance to salinity, drought, and root and leaf pathogens [28–30]. The study by Verma et al. (1998) indicated that various plants inoculated with *P. indica* exhibited increased biomass in both their aerial parts and roots compared to uninoculated controls [30]. Additionally, this fungus is widely used to promote the biosynthesis of secondary metabolites in plant cells and HRs cultures. An increase in secondary metabolites production was observed in suspension cultures of *Lantana camara* and hairy root cultures of *Linum album* treated with *P. indica* [25,31].

Research has shown that arbuscular mycorrhizae and some endophytes affect the phytochemical profiles of *E. purpurea* and other plant species [32–35]. For instance, studies found that *Aspergillus niger*, *Fusarium oxysporum*, and yeast extract enhanced the total phenolic and flavonoid content in *E. purpurea* calli [36]. Additionally, chemical elicitors such as gibberellic acid and methyl jasmonate (MeJA) increased both growth and the content of compounds like chicoric acid, caftaric acid, alkylamides, anthocyanins, phenolics, flavonoids, and polysaccharides in *E. purpurea* callus. [37,38]. However, the effects of *P. indica* on the production of secondary metabolites in *E. purpurea* have not been reported to date.

Given the high medicinal value of chicoric acid, the objectives of this study were to enhance chicoric acid biosynthesis and investigate the expression profiles of genes involved in its pathway within a new biotechnological platform based on HRs culture in *E. purpurea,* optimizing the use of elicitors. Therefore, the effects of two elicitors derived from *P. indica* (CE and CF) on chicoric acid production in *E. purpurea* HR were assessed for the first time.

## Materials and methods

The components of the culture medium and the chicoric acid standard used in this study were obtained from Sigma (USA) and Merck (Germany) chemical companies.

### Induction of the hairy roots

**Fig 1:**
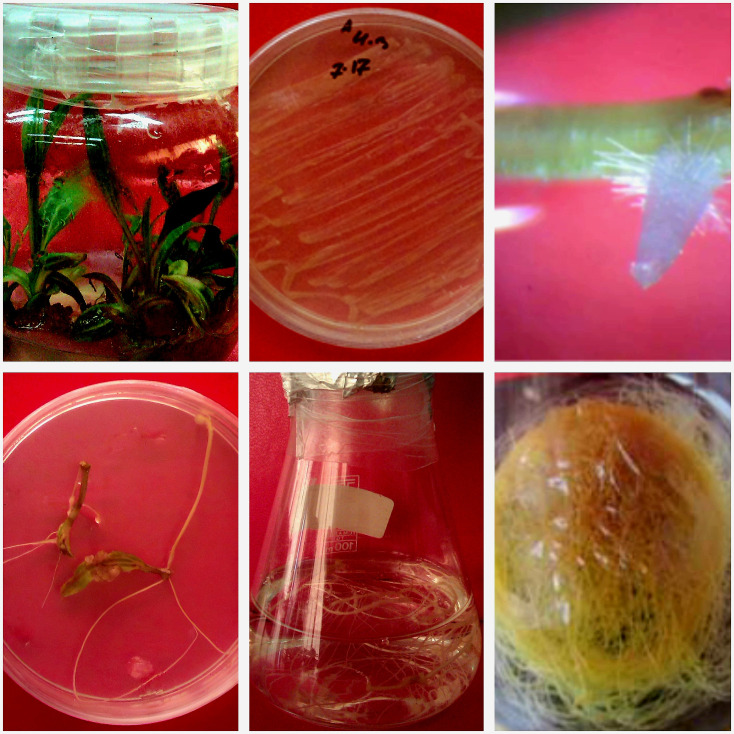
Seedling and hairy roots of *Echinacea purpurea.*

The seeds of *Echinacea purpurea* were sourced from Pakan Bazr Co. (http://www.pakanbazr.com). To sterilize the seeds, they were immersed in 70% (*v/v*) ethanol for 1 min and then in 5.25% (*v/v*) sodium hypochlorite for 20 min. Following this, the seeds were thoroughly washed with sterile distilled water. To produce seedlings of *E. purpurea*, the sterilized seeds were placed on Murashige and Skoog (MS) medium [39] and incubated in a growth chamber under a 16/8 h light/dark photoperiod at 25 ± 1°C (Fig 1).

*Agrobacterium rhizogenes* strain *AR*15834 was used in this study to induce hairy roots. This strain, which contains the *rolB* gene, was cultured in 10 ml of liquid Luria-Bertani (LB) medium, supplemented with rifampicin (25 mg/l) and kanamycin (50 mg/l) in darkness at 28°C for 48 h with shaking. The bacterial cultures (OD 600 nm) were centrifuged at 3000 rpm for 10 min at 4 °C, after which the supernatant was discarded. Then, 10 mL of MS medium was added to the resulting precipitate and shaken at 180 rpm for 2 h. Subsequently, leaf explants from 60-day-old plants were cut, infected with the bacterial suspension for 10 min, dried with autoclaved filter paper, and then transferred to the MS medium in darkness at 28°C for 48 h. Afterward, the seedlings were transferred to fresh media containing the same constituents, supplemented with 500 mg/l of cefotaxime. Successive transfers to MS medium with gradually decreasing concentrations of cefotaxime were performed every four days to eliminate bacteria from the culture medium [40].

Subsequently, bacteria-free HRs, measuring 4–5 cm in length, were separated and transferred to culture medium containing 150 mg/l of cefotaxime. After several sub-culturing, the bacteria-free HRs were transferred to 120 ml Erlenmeyer flasks containing 60 ml of cefotaxime-free liquid Woody Plant Medium (WPM) [41].

### Molecular confirmation and PCR analysis

DNA extraction from the HRs was performed using the cetyltrimethylammonium bromide (CTAB) method [42]. To confirm the presence of the *rolB* gene in the hairy roots, PCR analysis was conducted using specific primers for the *rolB* gene, as described by Chaudhuri et al. [43]. The sequences of the primers employed in the PCR were as follows: F *rolB*: 5′-GCTCTTGCAGTGCTAGATTT-3′; R *rolB*: 5′-GAAGGTGCAAGCTACCTCTC-3′. DNA was extracted from the transgenic roots, non-transformed roots (negative control), and *AR*15834 strain (positive control). After transgenic confirmation, the HRs were propagated and sub-cultured to perform the desired experiments.

### Measurement of HRs growth

Hairy roots (0.5 g) were cultured in a 100 ml flask containing 60 ml WPM medium (in three replicates) and incubated at 110 rpm and 25 °C in the dark. The roots were harvested every two days until the 30th day of the growth cycle. The HRs were separated from the liquid medium by filtration (Whatman No. 1), washed with distilled water to remove any residual medium, and then dried after freeze-drying to determine their dry weight (DW). The growth curve was plotted by graphing the DW of the hairy roots against time.

### Fungal elicitors

This study examined the effects of two types of elicitors derived from *Piriformospora indica*: culture filtrate (CF) and cell extract (CE), on the growth and chicoric acid production in the HRs cultures of *E. purpurea*. The elicitors were prepared following the protocol described by Baldi et al. (2009) with some modifications [44]. Fungal mycelia were cultured in 250 ml Erlenmeyer flasks containing 100 ml of potato dextrose broth (PDB) and incubated at 110 rpm at 30 ± 2 °C for 8 days (S1 Fig). The fungal biomass was separated by filtration. Then, the mycelia were washed three times with double-distilled water and freeze-dried to a constant weight. The dry cells were then ground in liquid nitrogen using a mortar and pestle. Finally, the crushed cells were mixed with water (3 mg/ml) and thoroughly blended. The resulting suspension was incubated at 90 °C for 60 min for hydrolysis with continuous shaking. After centrifugation at 10,000 × *g* for 15 min, the supernatant was collected and filtered through 0.22 μm cellulose acetate syringe filters and was designated as cell extract (CE) [45].

To obtain the culture filtrate (CF), the *P. indica* culture was filtered to separate the fungal biomass, and the culture broth was centrifuged at 10,000 × g for 20 min to remove all suspended particles. The mycelium-free medium was then filtered through a 0.22 μm membrane filter. The obtained transparent medium was designated as culture filtrate (CF) [45].

The HRs (0.5 g) were grown in 100 ml Erlenmeyer flasks containing 60 ml of WPM medium for elicitation. Three replicates were used for each HRs treatment. Various concentrations of the elicitors (1.25, 2.5, 5, and 10% *v/v*) were added to the HRs cultures during the late log-growth phase (on days 24 and 26). The flasks were incubated in triplicate at 110 rpm at 25 °C in the dark. After elicitation on day 24, the HRs were harvested on days 26 and 28 (48 and 96 h after elicitation, respectively). Following elicitation on day 26, the HRs were harvested only on day 28 (48 h post-elicitation).

Subsequently, the HRs were analyzed for biomass (dry weight), chicoric acid concentration, and molecular analysis. The untreated HRs cultures were used as control samples, containing equal volumes of PDB medium and water for CF and CE, respectively. In the case of HR biomass, as described above, DW was determined by freeze-drying the HR until the achievement of constant weight. The chicoric acid content was measured using high-performance liquid chromatography (HPLC), and molecular analysis was performed using real-time PCR.

### Extraction and determination of chicoric acid by HPLC

Hairy roots cultures (0.5 g) treated with different concentrations of fungal elicitor were harvested at the specified times. The chicoric acid content in *E. purpurea* HRs was quantified using HPLC analysis. The HRs samples were thoroughly ground in liquid nitrogen using a mortar and pestle after freeze-drying. The 20-mg powder samples were ultrasonicated for 30 min at room temperature in 1 ml mixed (70:30, *v/v*) aqueous methanol and 0.1% phosphoric acid in an ultrasonic water bath (LC 130H, Elma, Singen, Germany). The extracts were then centrifuged for 5 min at 10,000g, and the supernatant was filtered using 0.22 μm cellulose acetate syringe filters. The resulting extract was analyzed by HPLC (Waters, USA). The analysis of chicoric acid was performed using a C18 analysis column (Macherey-Nagel EC 250/4.6 Nucleodur). For each sample injection (20 μL), the mobile phase consisted of acetonitrile and phosphoric acid 0.1% (70:30 *v/v*), with a flow rate of 0.8 mL/min and detection at a wavelength of 230 nm. The content of chicoric acid was estimated using a calibration curve of standard chicoric acid. The reference standard of chicoric acid (purity >98%) was obtained from Sigma-Aldrich (Taufkirchen, Germany).

### Gene expression analysis

The gene expression levels of chicoric acid biosynthesis pathway were investigated at 3 and 12 h after adding the fungal elicitors to *E*. *Purpurea* HR culture by qRT-PCR using gene-specific primers and *β-actin* as a reference gene. Total RNA was extracted from 100 mg of both treated and untreated HRs samples using the RNX™-Plus solution kit (CinnaGen Inc, Tehran, Iran), following the manufacturer’s instructions. The quantity and quality of the extracted RNA were assessed using a Nanodrop spectrophotometer and 1% agarose gel electrophoresis. To remove genomic DNA contamination, the extracted RNA was treated with RNase-free DNase I (Fermentas, St. Leo-Roth, Germany) and verified through PCR using *β-actin* as the internal control gene. DNase-treated RNA (2 μg) was then used to synthesize cDNA using the reverse transcriptase enzyme RevertAid™ M-MuLV (Fermentas, Burlington, ON, Canada). The expression level of the *PAL*, *C3H*, *C4H*, *4CL*, and *HCT* genes was measured using specific primers (as detailed in S1 Table) through quantitative real-time PCR (qPCR) with the Applied Biosystem/MDS SCIEX system (Foster City, CA, USA). The qRT-PCR was conducted using SYBR Green Master mix (Biofact) on a 48-well platform system (Step One Plus device). PCR amplification included an initial denaturation at 95 °C for 10 min, followed by 40 cycles of 95 °C for 45 seconds, 60 °C for 20 seconds, and 72 °C for 25 seconds. All reactions were performed in three biological replicates, and the relative gene expression levels were calculated using the 2^-ΔCt^ method [46].

### Statistical analysis

The experiments were conducted as a factorial based on a randomized complete block design (RCBD) with three factors: the type of elicitor (two levels), the concentration of elicitor (four levels), and the timing of elicitor addition (two levels). Analysis of variance (ANOVA) and mean comparisons were conducted using the least significant difference (LSD) method with SAS software (SAS 9.1) at a significance level of P-values < 0.05. Graphs were created using GraphPad Prism 5 software.

## Results

### Induction of HRs

HRs were established using leaf explants induced by *A. rhizogenes* strain *AR*15834 containing Ri plasmid one month after infection (Fig 1). The PCR reaction with the DNA extracted from HR led to the amplification of a fragment with a length of 423 bp using *rolB* primer, which was equal to the size of this gene in the plasmid of strain *AR*15834 (positive control).

**Fig 2:**
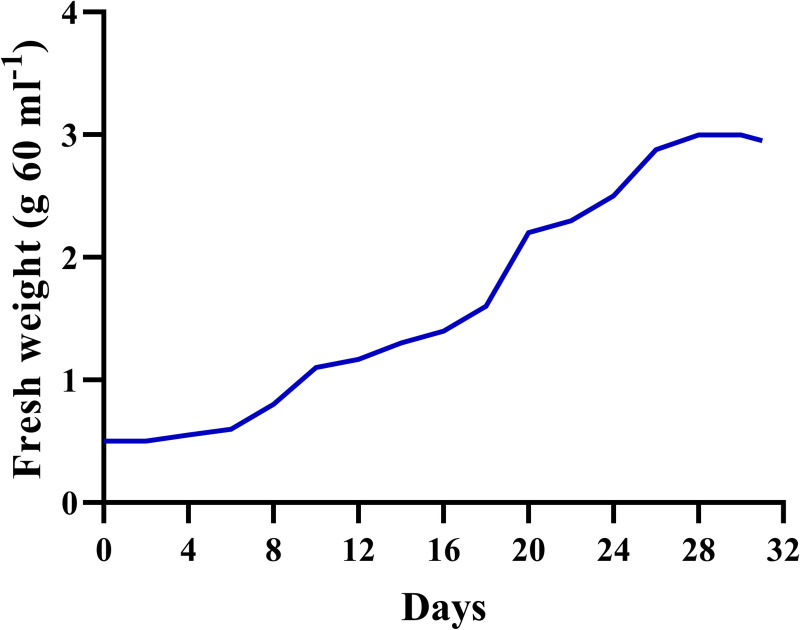
Cell growth curve based on dry weight in the hairy roots of *Echinacea purpurea.*

Based on *E. purpurea* HR growth curve (Fig 2), days 24 and 26 of the HR growth cycle were selected for elicitation.

### Effect of elicitor exposure time on total chicoric acid content

**Fig 3:**
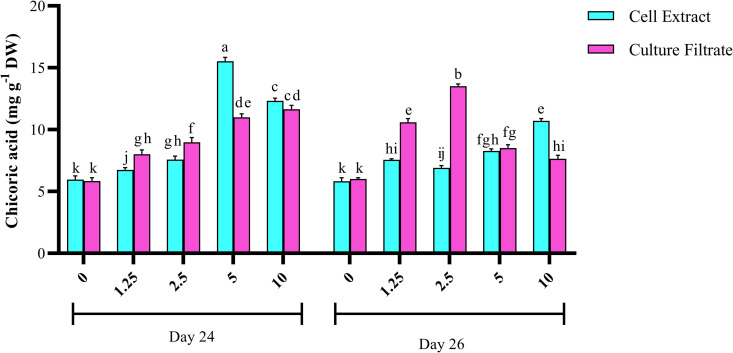
Effects of adding cell extract (CE) and culture filtrate (CF) of *P. indica* with different concentrations and addition time on chicoric acid production in *Echinacea purpurea.*

In HRs cultures exposed to 1.25% and 2.5% (*v/v*) of *P. indica* CE on the 24th day, no significant differences in chicoric acid production were observed between the samples harvested on the 26th and 28th days. However, when HR cultures were subjected to 5% and 10% (*v/v*) concentrations of *P. indica* CE on the 24th day, there was a significant decrease in chicoric acid production by the 28th day compared to the 26th day. Furthermore, the exposure of HRs cultures to different concentrations of *P. indica* CF on the 24th day showed significant effects on chicoric acid production (S2 Fig). HRs treated with various CF concentrations on the 24th day exhibited the highest chicoric acid content on the 26th day of the growth cycle. The optimal concentration of fungal elicitors for inducing the highest chicoric acid content in HRs culture was closely linked to the elicitation time. The highest content of chicoric acid in HR cultures exposed to different concentrations of CE and CF on the 24th day was obtained on the 26th day, compared with 28th day (Fig 3). Therefore, in the subsequent experiment, investigation of the effects of different concentrations of the elicitors and their adding times on chicoric acid production, the roots were merely harvested on 26th day.

### Effect of elicitor-addition time and concentration on chicoric acid content

The effects of CE and CF were evaluated on chicoric acid production in different concentrations and addition times. Chicoric acid yield was significantly affected by these combined treatments. The main effects of these factors and their interactions were significant (S2 Table) (Fig 3). Adding 5% and 10% (*v/v*) CE and CF to HRs cultures on the 24th day resulted in a significant enhancement of chicoric acid production compared to the lower concentrations of 1.25% and 2.5% (*v/v*). Notably, HRs cultures subjected to 1.25% and 2.5% (*v/v*) of CF on the 26th day displayed higher chicoric acid production than those subjected to 5% and 10% (*v/v*), (10.58 and 13.5 mg/g DW, respectively) which were about 1.8 and 2.29 fold of those of the controls (exposed with 1.25 and 2.5% % (v/v) of filter sterilized water on 26th day, respectively). Overall, all concentrations of CE and CF significantly improved chicoric acid production (Fig 3). The highest total yield of chicoric acid was obtained using 5% (*v/v*) CE on the 24th day of the culture cycle (15.52 mg/g DW), which was about 2.66 times of control (exposed with 5% (*v/v*) of filter sterilized water on 24th day).

The addition time of the elicitor significantly affected the production of chicoric acid in HRs cultures. On the 26th day, the optimal concentrations of both the CE and CF were determined to be 10% and 2.5% (v/v), respectively. Although similar results were observed with the addition of 1.25% and 2.5% (v/v) CE on the 26th day, a significant difference was noted between these two concentrations on the 24th day. The addition of 1.25% and 2.5% (v/v) CF on the 26th day was more effective than on the 24th day. Notably, elicitation with 5% and 10% (v/v) concentrations of both elicitors showed a high effect on chicoric acid production on the 24th day compared to the 26th day. Among the fungal elicitors, the addition of 5% (v/v) CE on the 24th day produced the highest results (15.52 mg/g DW), yielding 2.66 times more chicoric acid than the control. Furthermore, using 2.5% (v/v) CF on the 26th day resulted in the highest chicoric acid productivity (13.5 mg/g DW), being approximately 1.27% and 2.29% higher compared to the 24th day and the control, respectively.

### Effect of elicitor derived from *P. indica* on the growth rate of HRs

**Fig 4:**
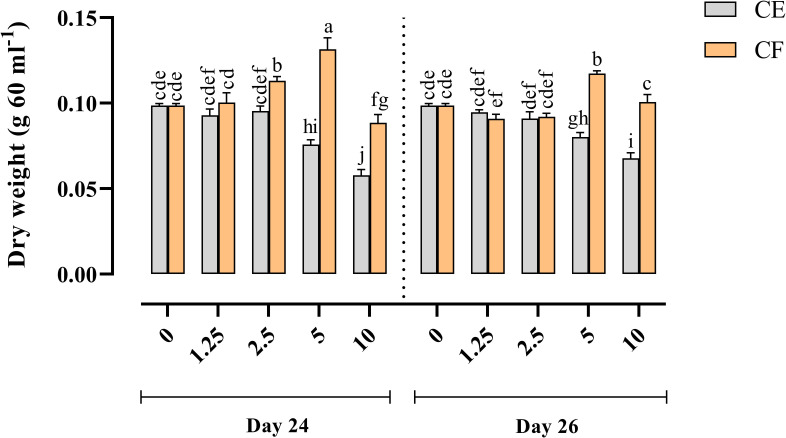
Effects of adding cell extract (CE) and culture filtrate (CF) of *P. indica* on HRs growth of *Echinacea purpurea.*

The effects of various concentrations of *P. indica* CE and CF, along with the timing of elicitor addition (age of HRs) and exposure time in the late log-growth phase, were assessed by measuring the dry weight of HRs cultures (Fig 4). The addition of *P. indica* CE to the HRs culture, regardless of concentration levels and addition time, significantly decreased HRs growth as compared to the control. In HRs cultures treated with CF on the 24th day, the highest significant DW was observed on the 28th day of the cell growth cycle. Consequently, for subsequent analyses, harvesting on the 28th day was selected.

The main effects of the type of elicitor, concentration, and their reciprocal interaction (excluding the triple interaction) were found to be significant (S2 Table). The overall effects of CE and CF on HRs growth indicated that the type of elicitor (CE or CF) significantly influenced the growth index of HRs cultures based on concentration, adding time, and exposure time (Fig 4). The most substantial growth reduction (1.59-fold lower than the control) was noted in HRs treated with a 10% (*v/v*) concentration of CE when added on the 24th day. A lower concentration (1.25% (*v/v*)) also resulted in decreased growth, but to a lesser extent (1.06-fold lower than the control). Cultures exposed to CF on days 24 and 26 showed an upward trend in HRs growth with an increase in elicitor concentration up to 5% compared to the control. However, HRs cultures treated with 10% (*v/v*) CF on the 24th and 26th days showed a growth reduction compared to those treated with 5% (*v/v*) CF. The highest growth index (1.36% increase compared to the control) (2.19 g/L) was recorded in HRs cultures exposed to 5% (*v/v*) CF on the 24th day, as addition time (harvested on the 28th day). HRs cultures treated with different concentrations of CF on the 26th day (and harvested on the 28th) exhibited lower DW compared to those treated on the 24th day due to the shorter time for the stimulating compounds of CF to induce HRs growth.

### Effect of CE and CF of P*. indica* on gene expression

**Fig 5:**
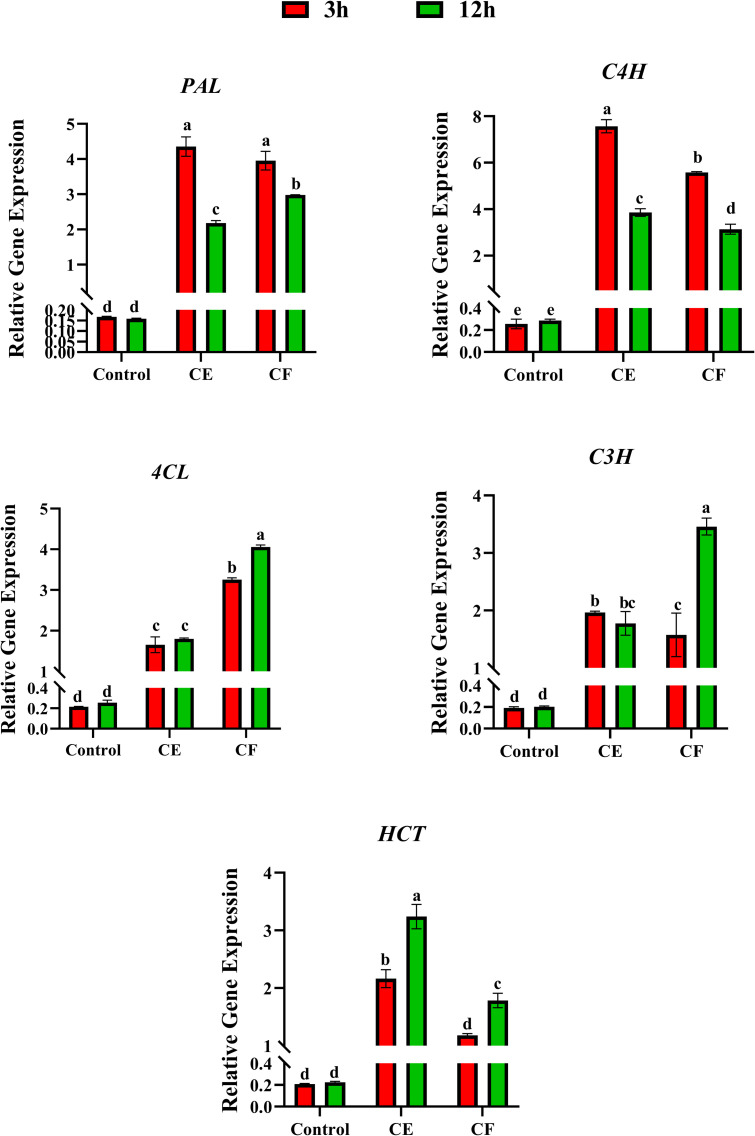
Expression analysis of genes involved in the biosynthetic pathway of chicoric acid of *Echinacea purpurea.*

Quantitative RT-PCR was performed to evaluate the expression levels of genes involved in the chicoric acid biosynthesis pathway, including *PAL*, *C4H*, *C3H*, *4CL*, and *HCT*. These genes were examined in *E. purpurea* HRs cultures treated with 5% (*v/v*) CE and 2.5% (*v/v*) CF of *P. indica*, as these treatments produced the highest amounts of chicoric acid in the HRs culture. The results indicated that the expression levels of all genes were enhanced in the HRs cultures treated with both elicitors at all studied times (3 and 12 h after elicitation) compared to the control. Notably, a strong induction of gene expression occurred with the 5% (*v/v*) CE, with the highest expression observed for the *C4H* and *PAL* genes (29.64 and 26.2-fold obtained in the control culture, respectively) (Fig 5). In HRs cultures treated with 5% (*v/v*) CE, the highest expression levels of *C4H* and *PAL* were detected 3 h after elicitation, while *HCT* gene expression peaked at 12 h post-elicitation (14.48-fold obtained in the control culture). No significant differences were observed in the expression levels of *C3*H and *4CL* at 3 and 12 h after elicitation. Overall, the highest expression level in elicitor CE was related to the *C4H* gene at 3 h, with a 29.64-fold increase compared to the control cultures.

Additionally, all genes exhibited upregulation in HRs cultures elicited with 2.5% (*v/v*) CF. Our results demonstrated that the highest gene expression was related to *C4H* and *PAL* at 3 h after elicitation (23.82 and 21.88-fold obtained in the control culture), while the C3H and 4CL genes exhibited peak expression 12 h after the addition of 2.5% (*v/v*) CF (17.07 and 15.9-fold obtained in the control culture, respectively). It can be concluded that the upstream and downstream genes in the chicoric acid biosynthetic pathway had the highest expression both at 3 h and 12 h after elicitation, respectively (Fig 5).

Overall, our results indicate that the *PAL* gene had the highest relative expression in HRs treated with 5% (*v/v*) CE. The expression levels of the *PAL* gene in HRs cultures exposed to 5% (*v/v*) CE and 2.5% (*v/v*) CF at 3 h were higher compared to 12 h post-treatment. The expression pattern of the *C4H* gene, which is an upstream gene, was similar to *PAL* gene, with peak expression observed 3 h after adding 5% (*v/v*) CE. Transcript levels of the *4CL* gene reached their maximum at 12 h after adding 2.5% (*v/v*) CF to the cultures. Although the *C3H* gene expression increased in cultures exposed to 5% (*v/v*) CE compared to the control, there were no significant differences across different time points. The highest expression level of the *C3H* gene occurred 12 h after adding 2.5% (*v/v*) CF. The *HCT* gene demonstrated peak expression levels in cultures treated with both 5% (*v/v*) CE and 2.5% (*v/v*) CF at 12 h post-elicitation (Fig 5).

## Discussion

The production of phenolic compounds with antioxidant activity is one of the key defense mechanisms that plants use against stress [47]. *E. purpurea* HRs culture, known for its high growth rate, is a useful resource for producing chicoric acid as a phenolic compound [48]. Elicitors are effective methods for enhancing the production of valuable metabolites in HRs cultures [49,50]. They stimulate the plant’s defense system, improve the production of secondary metabolites, and can influence the growth rate [51]. One of the effective elicitors that enhances secondary metabolite production in plant cell culture is the fungal elicitor [52,53]. The effect of fungal elicitor on the production of bioactive compounds depends on various parameters, such as fungal species, elicitor type, elicitor concentration, incubation period of plant cells and elicitor, culture age, and medium nutrient composition [54–57]. In this study, we explored the effects of fungal elicitors derived from *P. indica* on HRs growth and chicoric acid production in *E. purpurea* for the first time. We employed two types of fungal elicitors (CE and CF) at various concentrations and at various addition times. These factors resulted in different responses concerning chicoric acid production and HRs growth rate.

The *P. indica* CE at concentrations of 5% and 10% (*v/v*) consistently reduced the growth of *E. purpurea* HRs across all addition times. This decline may be attributed to a decrease in primary metabolism and a shift towards secondary metabolite production [52,58]. Our findings are consistent with previous research indicating reduced cell growth in *Corylus avellana* cell suspension cultures after applying the CE from *Chaetomium globosum* [45]. Similar effects have been observed with fungal elicitors inhibiting cell growth in *Taxus chinensis* [24,52].

Additionally, the observed reduction in HR growth may also result from the toxic effects of CE, especially at higher concentrations. This is consistent with previous studies showing that a high concentration of the fungal elicitor *Rhizopus stolonifer* (50 mg/l) caused the highest rate of cell death in *Taxus baccata* cultures compared to lower concentrations [59]. A prior study noted a reduction in HRs growth of *Linum album* cultures exposed to the cell wall of *P. indica*, which supports our findings [53]. Other reports have similarly demonstrated that plant cell growth is often suppressed in cultures treated with various elicitors [60,61].

With increasing concentration of CF elicitor to 5%, a significant increase in HRs growth was observed on days 24 and 26. This may be due to the beneficial compounds present in the CF. It has been reported that the growth of cell suspension [62] and HRs of *L. album* [31,53] were significantly improved by elicitors derived from *P. indica*. Therefore, it can be hypothesized that the plant growth-promoting effect of *P. indica*, observed in the present study, might be due to the metabolites released into the medium during the growth of fungal cells. It has been reported previously that the addition of CF of *P. indica* promoted the growth of cell suspension [62] and HRs cultures in *L. album* [31]. In most studies, the growth-promoting effects of *P. indica* CF have been reported on *in vivo* and *in vitro* plants [63,64]. According to previous studies, endophytes typically do not induce strong hypersensitive reactions in their host plants, and some even promote plant growth [65]. However, distinct responses occur when cells are exposed to toxic compounds from endophytes [66]. In our study, a reduction in growth was observed in HRs cultures exposed to 10% (*v/v*) CF.

The use of different fungal elicitors derived from *P. indica*, with varying concentrations and exposure times, led to distinct responses in chicoric acid production in this study. Specifically, when *E. purpurea* HRs were exposed to 5% (*v/v*) CE on the 24th day and 2.5% (*v/v*) CF on the 26th day, the highest content of chicoric acid was produced. The previous studies [52,53,67] reported that the type, concentration, and adding time of elicitors affect the elicitation of secondary metabolites.

Our findings demonstrated that CE from *P. indica* is the most effective elicitor for chicoric acid biosynthesis in *E. purpurea* HRs. Overall, the interaction between plant cells and fungi triggers specific responses that enhance the production of secondary metabolites [68]. Plant cells possess receptors on their plasma membrane that recognize fungal elicitors and activate the plant cell defense system [69]. The recognition of elicitors by these receptors is the initial step in activating the defense mechanisms. The specificity of the receptor structure allows for the precise recognition of particular elicitors [70]. Therefore, not all fungal elicitors can effectively stimulate a cell culture system, making it essential to screen various elicitors for optimal production of desired compounds in a specific system. In our study, the lowest chicoric acid levels were observed at the lower elicitor concentration of 1.25%, regardless of exposure time. This suggests that at lower concentrations than optimal, not all binding sites in the cells may be occupied, which prevents the activation of chicoric acid biosynthesis. Conversely, a higher concentration (10%) may induce a hypersensitive response that leads to cell death. Therefore, an optimal elicitor concentration is needed to induce chicoric acid biosynthesis.

Different concentrations of CE and CF elicitors increased chicoric acid production 48 h after treatment (on the 26th day). However, when HRs were exposed to the elicitor for a longer duration (96 h), there was a decrease in chicoric acid production. Thus, regardless of the concentration of the elicitor, an exposure period of 48 h was identified as the optimal duration for significantly enhancing chicoric acid production (Fig 5).

Additionally, longer exposure times than 48 h might reduce chicoric acid production due to potential toxicity, or although higher chicoric acid synthesis may occur, final production could decline due to degradation. The growth phase of the HRs culture influences the response to the elicitor and the pattern of phytochemicals production. Therefore, the optimal timing for elicitor application depends on both the growth phase and the duration of exposure. Previous studies indicated that the content of podophyllotoxin and lariciresinol in *Linum album* cells treated with fungal elicitors varied based on the timing of elicitor addition, with the peak concentrations observed on the fifth day after treatment [71]. In another report [38], polyphenols and chicoric acid contents in *E. purpurea* cell suspensions exposed to MeJa significantly increased at five days post-elicitation compared to seven days. According to our findings, extended elicitation times (beyond 48 h) resulted in a decline in chicoric acid production, which was positively correlated with root biomass. In a report [72], the optimized exposure time of *L. album* HRs cultures with *P. indica* CF for increased lignan production was 24 h. According to the previous reports on different metabolites [44,45,52,53,71], which confirm our results, it can be concluded that to obtain the highest elicitation of chicoric acid production, it is necessary to optimize the concentration and addition time of the elicitor to the HRs culture. The elicitation mechanism of *P. indica* is still not well-known. However, it has been suggested that *P. indica* CE and CF, along with other fungal compounds such as enzymes, chitin, and disaccharides, act as signaling molecules to induce plant immune responses and initiate the phenylpropanoid biosynthesis pathway, thereby triggering the production of secondary metabolites such as phenolic and flavonoid compounds [44,53].

Induction of defense-related genes, leading to the accumulation of phenolic compounds, has been observed during early stress responses [73]. The variety of fungal elicitors can cause fluctuations in the production of phenolic compounds by altering gene expression and aromatic amino acid content, which is consistent with our results [53,67]. Research on the chicoric acid biosynthesis pathway has identified that *C4H* and *HCT* genes play an important role in the biosynthesis of this valuable secondary metabolite [74]. Therefore, the increased chicoric acid production could result from the increased expression of *C4H* and *HCT* genes. It has been demonstrated that UVB radiation upregulates the expression of *CHS*, *C4H*, and *CHI* genes in *Petunia axillaris*, leading to increased flavonoid production. Conversely, reducing *HCT* gene expression via the antisense technique significantly decreases lignin production in alfalfa and rice [75,76]. It has already been shown that *C3H* expression was improved significantly by MeJa, which correlated with the enhanced biosynthesis of chicoric acid in *E. purpurea* cell suspension. However, the expression of *PAL*, *4CL*, and *HCT* genes was independent of MeJa provision in *E. purpurea* cell suspension [75]. Elicitors can alter the expression profiles of genes involved in the biosynthetic pathways of metabolites through changes in hub IncRNA gene expression. A recent study [77] indicated that hub IncRNAs, such as CSE and BEBT, enhanced the expression of secondary metabolite biosynthesis pathway genes in *E. purpurea* under the influence of methyl jasmonate. Thus, fungal elicitors may induce changes in the expression profiles of chicoric acid pathway genes in the hairy roots of Echinacea by increasing the expression of hub RNAs.

According to our results, the increased expression of genes involved in the chicoric acid biosynthesis pathway, especially *PAL* and *C4H*, contributed to a greater expression of enzymes related to chicoric acid biosynthesis, ultimately leading to more chicoric acid biosynthesis in the HRs cultures, which is also consistent with the results of previous studies.

## Conclusion

In this study, two elicitors derived from *P. indica* (CE and CF) influenced HRs growth and chicoric acid accumulation, depending on the concentration and exposure time. Among the elicitors evaluated, the addition of 5% (*v/v*) CF on the 24th day of HRs culture resulted in the highest dry weight of *E. purpurea* HRs. Additionally, the use of 5% (*v/v*) CE on the same day resulted in the highest stimulation of chicoric acid biosynthesis.

Overall, CE proved to be a potent biotic elicitor for enhancing chicoric acid production. These findings were corroborated by the expression profiles of chicoric acid biosynthetic genes (*PAL*, *C4H*, *C3H*, *4CL*, and *HCT*). In conclusion, the results suggest that HRs cultures of *E. purpurea*, when amended with biotic elicitors, offer new insights into the production of valuable biochemical compounds.

## Supporting information

S1 Table Sequences of primers used for gene expression analysis. (DOCX)

S2 Table Analysis of variance for the effects of adding cell extract and culture filtrate of *Piriformospora indica* on the 24th and 26th days of the culture cycle on growth and Chicoric acid production of *Echinacea Purpurea* hairy roots. (DOCX)

S1 Fig *Piriformospora indica* in PDB medium. (TIF)

S2 Fig Analysis of the addition of the elicitors derived from *P. indica* (CF, and CE) on the 24th day, with two different harvesting days, on the content of chicoric acid. (TIF)
