# Supplementary material for: Piriformospora indica culture filtrate and cell extract induce chicoric acid production in Echinacea purpurea hairy roots
Source: PLoS One. 2025 Jun 17;20(6):e0323961. doi: 10.1371/journal.pone.0323961 (PMC12173382; doi:10.1371/journal.pone.0323961)
Supplement: S1 Table — (DOCX) [file pone.0323961.s001.docx]

**Supplementary Information**

***Piriformospora indica* culture filtrate and cell extract induce growth and chicoric acid production in *Echinacea purpurea* hairy roots**

Samane Khalili^1^, Ahmad Moieni^1^, Naser Safaie^2^, Mohammad Sadegh Sabet^1^

^1^ Department of Plant Genetics and Breeding, Faculty of Agriculture, Tarbiat Modares University, Tehran, P.O. Box: 14115-336, Iran

^2^ Department of Plant Pathology, Faculty of Agriculture, Tarbiat Modares University, Tehran, P.O. Box: 14115-336, Iran

| **S1 Table.** Sequences of primers used for gene expression analysis. | | |
| --- | --- | --- |
| **Primer Name** | **Primer Sequence (5`→3`)** | **PCR Product Length (bp)** |
| *ß Actin* (F) | AGGTTCTCTTCCAGCCATCTT | 160 |
| *ß Actin* (R) | TCTGTCAGCAATACCAGGGAA |  |
| *4CL* (F) | CGTCGTAGCTCTGCCGTACTC | 154 |
| *4CL* (R) | CAACACGCACATCAACACATC |  |
| *HCT* (F) | ATGGGTGGGCGGTTGAAGAGAGA | 112 |
| *HCT* (R) | CACCAAAATCATCCACAACACCGT |  |
| *PAL* (F) | TAAGCATCACCCGGGCCAAATAG | 195 |
| *PAL* (R) | ATTTTGGTGGCGAAGCGAATGAC |  |
| *C4H* (F) | GTCCAAATCACCGAGCCTGATG | 142 |
| *C4H* (R) | GGATGTCAAACCCACCGAGCTT |  |
| *C3H* (F) | CGAGCATGTTATGGGGTTGTAGTA | 160 |
| *C3H* (R) | GCATATCTCGCTTCCTTCCTTGTAG |  |

| **S2 Table.** Analysis of variance for the effects of adding cell extract and culture filtrate of *Piriformospora indica* on 24th and 26th days of culture cycle on growth and Chicoric acid production of *Echinacea Purpurea* hairy roots. | | | |
| --- | --- | --- | --- |
|  |  | Mean squares | |
| Source of variation | Degree of freedom | Dry weight | Chicoric acid (μg g^-1^ DW) |
| Block | 2 | 0.000055^ns^ | 0.1315 ^ns^ |
| Elicitor type (A) | 1 | 0.0048^**^ | 2.059^**^ |
| Concentration level (B) | 4 | 0.00097^**^ | 49.002^**^ |
| elicitor-adding time (C) | 1 | 0.000064^ns^ | 11.141^**^ |
| A × B | 4 | 0.0012^**^ | 19.411^**^ |
| A × C | 1 | 0.00029^**^ | 14.617^**^ |
| B × C | 4 | 0.00022^**^ | 25.143^**^ |
| A × B × C | 4 | 0.000068^ns^ | 8.425^**^ |
| Error | 38 | 0.000032 | 0.208 |
| *, ** and ns indicate significant difference p<0.05, significant difference p<0.01 and non-significant, respectively. | | | |


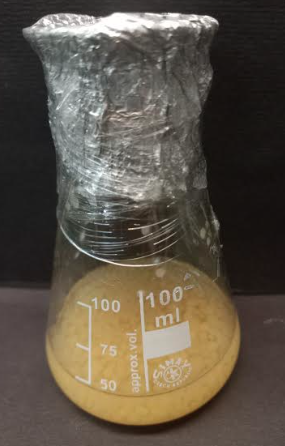


**S1 Fig.** *Piriformospora indica* in PDB medium.

**
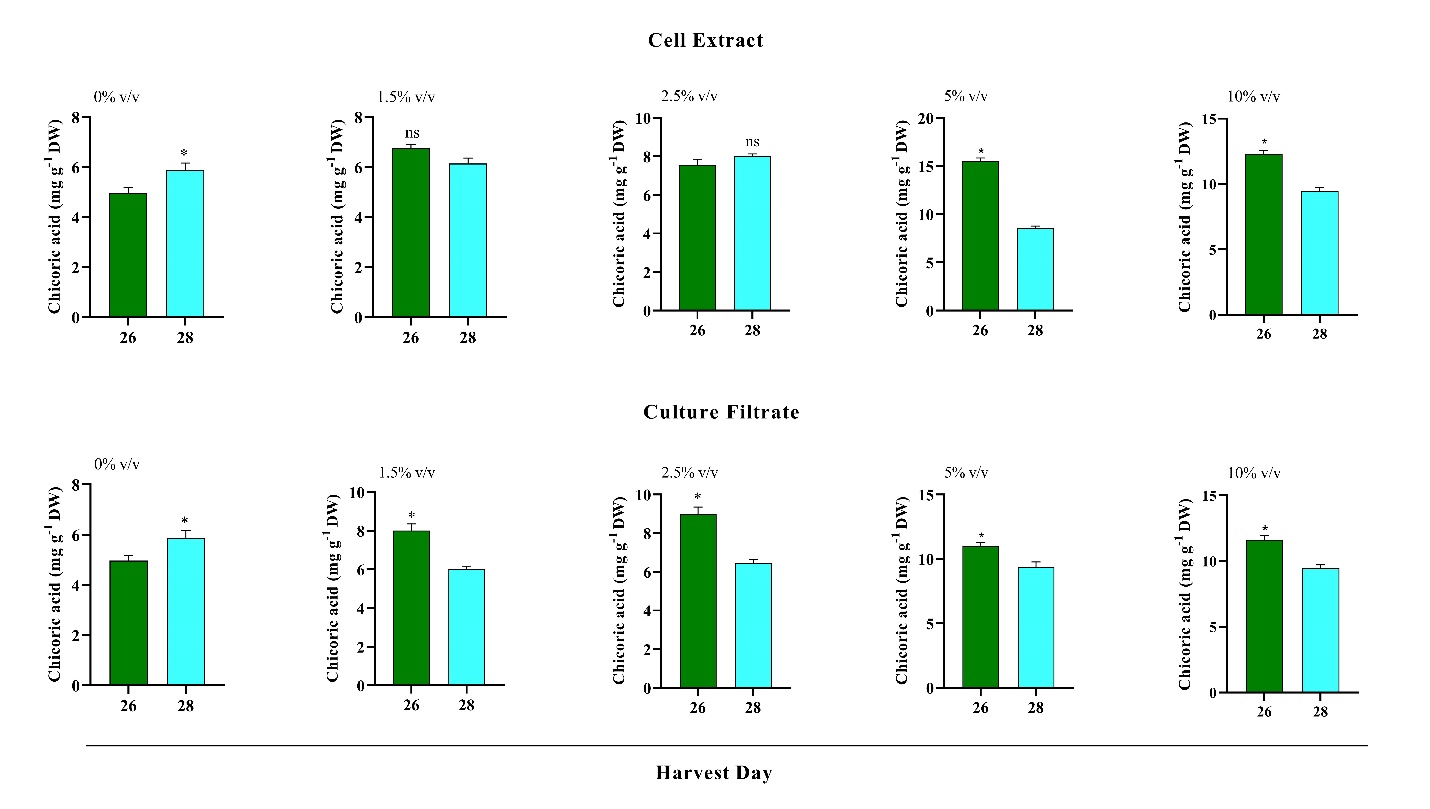
**

**S2 Fig.** Analysis of addition on the 24th day with two different harvesting days on the content of chicoric acid (* and ns indicate significant differences *p*<0.05 and non-significant, respectively)
