# Supplementary figures and images for: Piriformospora indica culture filtrate and cell extract induce chicoric acid production in Echinacea purpurea hairy roots
Source: PLoS One. 2025 Jun 17;20(6):e0323961. doi: 10.1371/journal.pone.0323961 (PMC12173382; doi:10.1371/journal.pone.0323961)

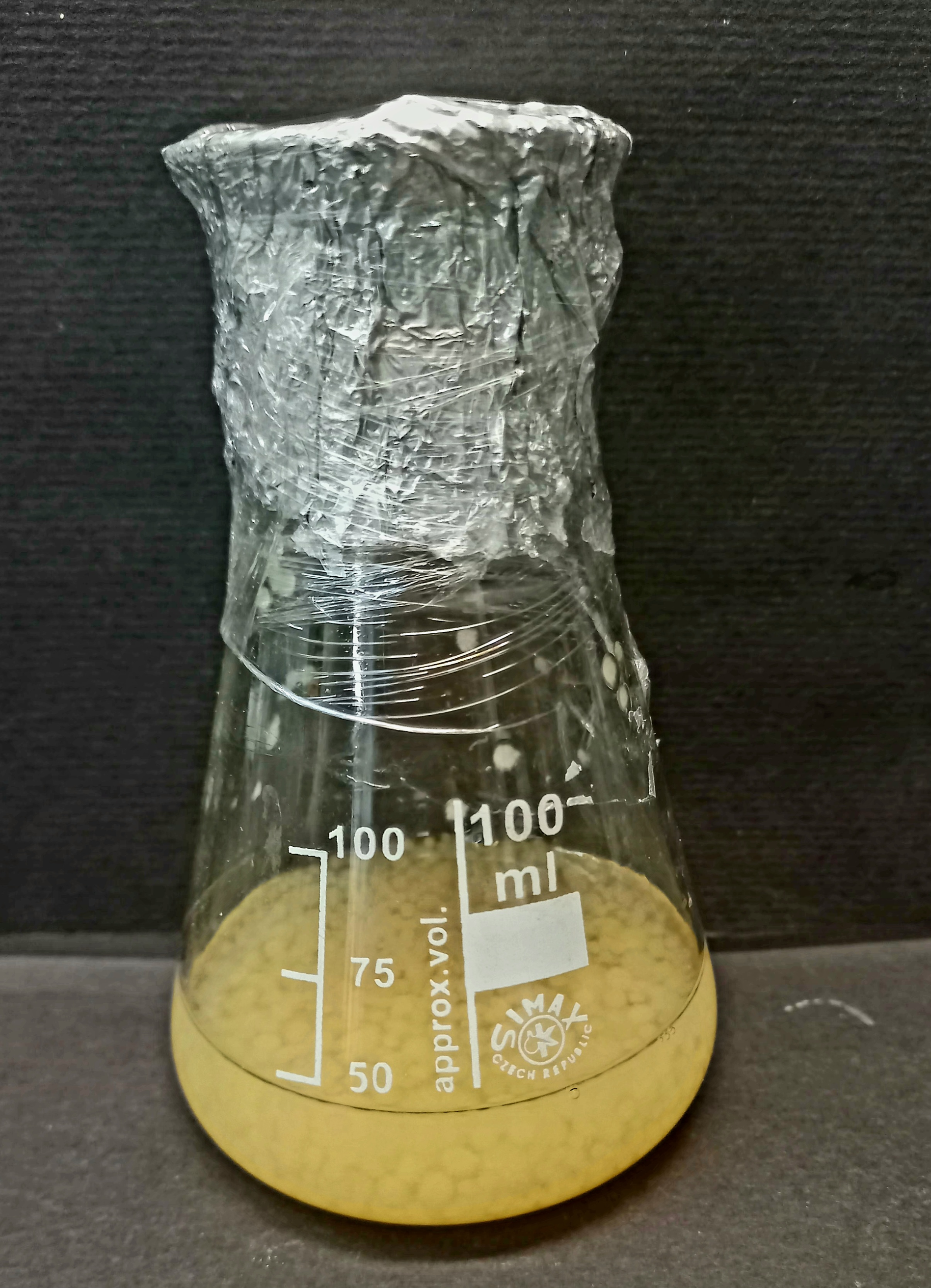

Supplement: S1 Fig — (TIF) [file pone.0323961.s003.tif]

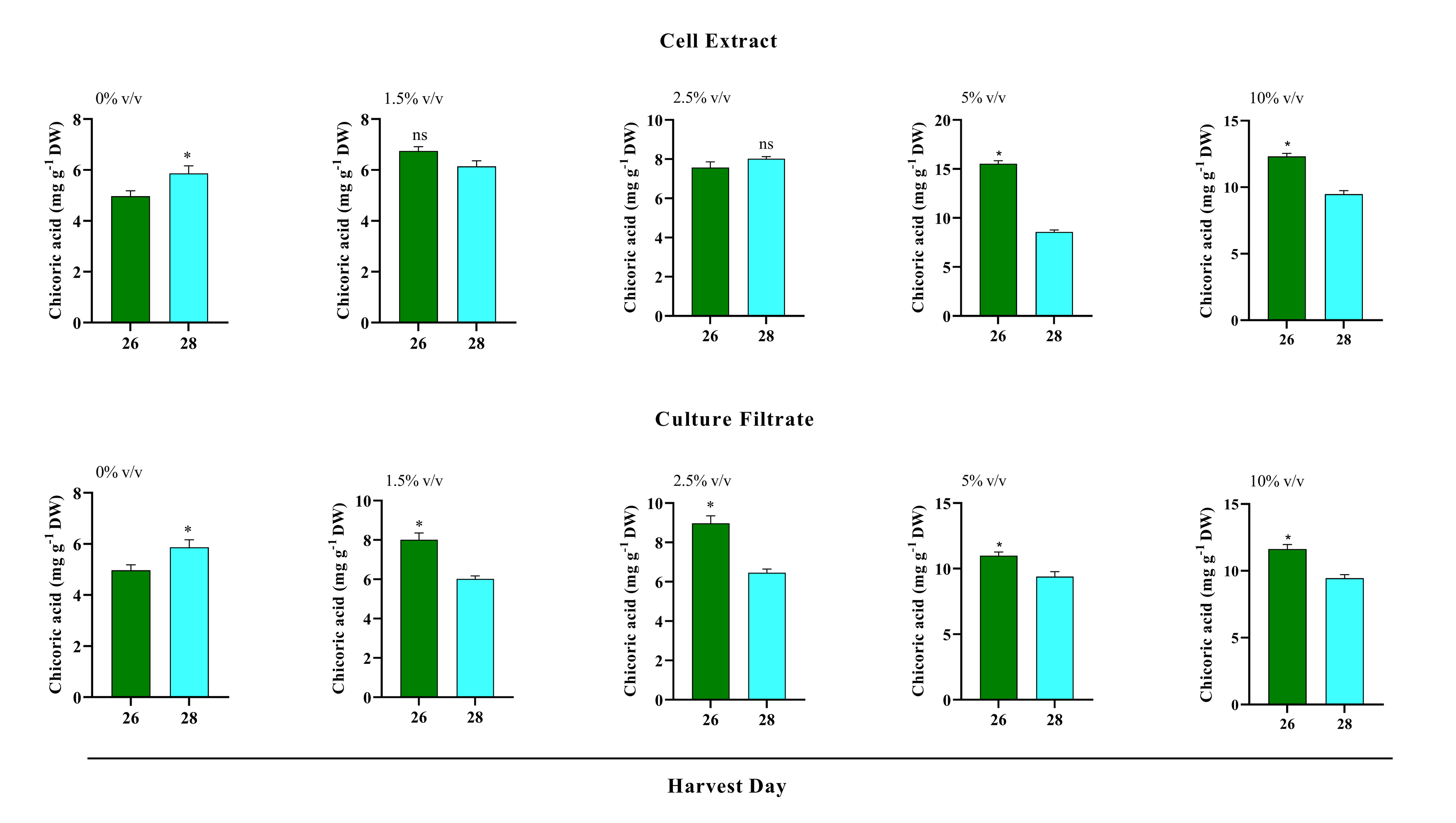

Supplement: S2 Fig — (TIF) [file pone.0323961.s004.tif]
